# RNA splicing regulator EIF3D regulates the tumor microenvironment through immunogene-related alternative splicing in head and neck squamous cell carcinoma

**DOI:** 10.18632/aging.205681

**Published:** 2024-03-25

**Authors:** Dandan Lu, Mijti Mihoayi, Yimin Ablikim, Abdeyrim Arikin

**Affiliations:** 1 Otolaryngology Diagnosis and Treatment Center, People’s Hospital of Xinjiang Uygur Autonomous Region, Urumqi 830000, China; 2 Department of Otolaryngology, Shaanxi Nuclear Industry 215 Hospital, Xianyang 712000, China

**Keywords:** HNSC, EIF3D, tumour microenvironment, immunogene-related alternative splicing, immunotherapy

## Abstract

Study finds that eukaryotic translation initiation factor 3 subunit D (EIF3D) may play an important role in aberrant alternative splicing (AS) events in tumors. AS possesses a pivotal role in both tumour progression and the constitution of the tumour microenvironment (TME). Regrettably, our current understanding of AS remains circumscribed especially in the context of immunogene-related alternative splicing (IGAS) profiles within Head and Neck Squamous Cell Carcinoma (HNSC). In this study, we comprehensively analyzed the function and mechanism of action of EIF3D by bioinformatics analysis combined with *in vitro* cellular experiments, and found that high expression of EIF3D in HNSC was associated with poor prognosis of overall survival (OS) and progression-free survival (PFS). The EIF3D low expression group had a higher degree of immune infiltration and better efficacy against PD1 and CTLA4 immunotherapy compared to the EIF3D high expression group. TCGA SpliceSeq analysis illustrated that EIF3D influenced differentially spliced alternative splicing (DSAS) events involving 105 differentially expressed immunogenes (DEIGs). We observed an induction of apoptosis and a suppression of cell proliferation, migration, and invasion in EIF3D knock-down FaDu cells. RNA-seq analysis unveiled that 531 genes exhibited differential expression following EIF3D knockdown in FaDu cells. These include 52 DEIGs. Furthermore, EIF3D knockdown influenced the patterns of 1923 alternative splicing events (ASEs), encompassing 129 IGASs. This study identified an RNA splicing regulator and revealed its regulatory role in IGAS and the TME of HNSC, suggesting that EIF3D may be a potential target for predicting HNSC prognosis and immunotherapeutic response.

## INTRODUCTION

Head and neck cancer ranks among the most prevalent malignancies globally [1], with squamous cell carcinoma accounting for over 90% of head and neck cancer cases [2]. In recent years, patients afflicted with Head and Neck Squamous Cell Carcinoma (HNSC) have undergone various treatment modalities, including surgery, chemotherapy, radiotherapy, and immunotherapy. However, the clinical outcomes were proved unsatisfactory, underscoring the pressing need to identify effective molecular targets and potential therapeutic methods.

Eukaryotic initiation factor 3d (EIF3D) is a recognised RNA-binding subunit of the eukaryotic initiation factor-3 (eIF3) complex weighing 66 to 68 kDa, featuring an RNA-binding motif and a cap-binding domain [3]. Investigations of human cancer tissue consistently exhibited that EIF3D increased expression with an unfavorable prognosis in gastric, gallbladder, lung, and liver cancers [4–7]. Interestingly, in the context of prostate cancer, an increased EIF3D expression correlates with a more positive prognosis [8]. The underlying reasons for these discrepancies remain unclear and may be linked to different cancer types. The association between EIF3D and prognosis, whether positive or negative, implies its potential involvement in disease aggressiveness, progression, or therapeutic response. Multiple studies also illustrated EIF3D’s pivotal role in carcinogenesis through the regulation of translational initiation and oncogene expression [4, 9–12], suggesting the potential utility as both a novel biomarker and a target for cancer therapeutics. Nonetheless, it remains imperative to ascertain whether EIF3D expression levels correlate with pathological characteristics and disease progression and how EIF3D recognises and regulates translation, particularly in the context of dysregulated expression in HNSC.

The translation process can be categorised into initiation, elongation, termination, and ribosome recycling, with initiation as a pivotal step. Among these stages, translation initiation stands out as one of the most critical phases in the translation process. For the majority of cellular mRNAs, the initial step of mRNA translation involves the recognition of the 5′ 7-methylguanosine (m7G) cap by the eukaryotic initiation factor 4E (EIF4E), a constituent of the heterotrimeric EIF4F complex. Subsequently, the 5′ cap-bound EIF4F recruits the small (40S) ribosomal subunit, accompanied by various translation initiation factors, thereby facilitating the efficient translation of eukaryotic mRNAs [13]. Nevertheless, certain mRNAs undergo translation in a cap-independent manner. These capped mRNAs do not rely on EIF4E and are translated under various cellular conditions, including where EIF4E activity is compromised, such as during cellular stress states [14], viral infections [15], and in diseases like cancer [4]. Notably, Lee et al. demonstrated that EIF3D binding in the position 67 to 153 within the 5′-UTR of c-Jun mRNA may obstruct EIF4F binding to the 5′ end of the 5′-UTR. Deletion of the EIF3D-binding sequence could effectively negate EIF3D’s inhibitory effect on EIF4F binding [9, 16]. Furthermore, EIF3D’s role in translation initiation extends to the mRNA encoding the core U2 spliceosomal component protein SF3A3 during Myc-driven oncogenesis. This function of EIF3D is contingent upon a stem–loop structure present in the 5′-UTR of SF3A3 mRNA [11], implying a reciprocal regulatory relationship between EIF3D and the splicing factor SF3A3 in driving the noncanonical cap-dependent translation process.

Alternative splicing (AS) represents a pervasive mechanism for regulation of gene expression in eukaryotic transcripts, contributing substantially to the diversification of gene and protein functions through intron removal and exon ligation in pre-mRNAs [17]. Notably, AS has been associated with the heterogeneity of the TME. The TME constitutes of highly heterogeneous and dynamic network that exerts influence over tumour initiation and progression. It achieves this by facilitating cancer cell survival, migration, metastasis, chemoresistance, and evasion of immune responses, particularly in response to immunosuppressive agents like programmed cell death protein 1 (PD1) and cytotoxic T-lymphocyte-associated antigen 4 (CTLA4) [18–20]. Recent research has illuminated the significant role of AS in governing the TME, particularly in the context of immunogene-related alternative splicing (IGAS) events [21, 22]. These AS-influenced immune processes have garnered substantial attention, encompassing pathways such as the B cell receptor signaling pathway, T cell receptor signaling pathway, and natural killer cell cytotoxicity [23]. IGAS augments mRNA diversity during transcription, potentially exerting an impact on the TME, thereby influencing the development of immunotherapeutic strategies against cancer. Nevertheless, it remains to be elucidated whether EIF3D regulates AS in a manner that mediates disparities in the tumour microenvironment.

In this study, we combined bioinformatics analysis with *in vitro* experiments to uncover the role of EIF3D as an AS regulator. Our findings suggest that EIF3D may impact the heterogeneity of the TME by modulating IGAS events, consequently influencing the progression of Head and Neck Squamous Cell Carcinoma (HNSC) and its responsiveness to immunotherapy. This study significantly broadens our understanding of EIF3D’s functions and offers valuable insights for the development of innovative therapeutic strategies for HNSC.

## MATERIALS AND METHODS

### Data collection and analysis

First, we acquired mRNA expression data for EIF3D from the TIMER database (https://cistrome.shinyapps.io/timer/) encompassing both pan-cancer cohorts and samples from normal individuals. Additionally, transcriptome sequencing data, along with corresponding clinical information for 522 HNSC samples and 44 normal samples, were procured from The Cancer Genome Atlas (TCGA) database (https://portal.gdc.cancer.gov). Subsequently, we categorised HNSC patients into EIF3D high-expression group and the EIF3D low-expression group based on the median EIF3D expression level. To describe differences in the composition of the TME between these groups, we employed ESTIMATE to compute ImmuneScores, StromalScores, and ESTIMATEScores. Furthermore, we conducted an analysis of immune cell infiltration within the tumour based on CIBERSORT software (https://cibersort.stanford.edu/). In order to compare immuno-therapy scores between the high and low expression EIF3D groups, we obtained immuno-therapy scores for samples within the TCGA database from TCIA (https://tcia.at), specifically for anti-CTLA4 and anti-PD1 inhibitor treatments.

For a comprehensive understanding of mRNA alternative splicing (AS) patterns, we retrieved detailed AS data from TCGA SpliceSeq (https://bioinformatics.mdanderson.org/TCGASpliceSeq/). This data encompassed seven primary AS event types, including Alternate Acceptor site (AA), Alternate Donor site (AD), Alternate Promoter (AP), Alternate Terminator (AT), Exon Skipping (ES), Mutually Exclusive Exons (ME), and Retained Intron (RI). AS patterns of protein-coding genes in HNSC samples were delineated based on specific criteria, requiring a Percent Spliced In (PSI) value of ≥75% and a minimum standard deviation of ≥0.10. To delve deeper into the role and underlying mechanisms of EIF3D in HNSC, this study subsequently conducted a series of *in vitro* cellular experiments.

### Lentivirus information

We obtained all siRNA duplexes from Genepharma (Suzhou, China). The non-targeting control siRNA had the following sequence: 5′-UUCUCCGAACGUGUCACGUTT-3′ (sense). The siRNA targeting EIF3D (siEIF3D) featured the following sequence: 5′-CCUAGAAUACUACGACAAATT-3′ (sense).

### Cell culture and transfections

FaDu cell line (CL-0083, Procell Life Science and Technology Co., Ltd., China) was maintained in MEM (PM150410, Procell Life Science and Technology Co., Ltd., China) with 10% fetal bovine serum (FBS) (10091148, Gibco, China), 100 μg/mL streptomycin, and 100 U/mL penicillin (SV30010, Hyclone, USA) at 37°C with 5% CO_2_. Transfection of siRNA into the cells was carried out using Lipofectamine™ RNAiMAX Transfection Reagent (13778030, Invitrogen, Carlsbad, CA, USA) following the manufacturer’s instructions. After 48 hours, transfected cells were collected for RT-qPCR analysis.

### Quantitative real-time PCR

Glyceraldehyde-3-phosphate dehydrogenase (GAPDH) served as the control gene for assessing the impact of EIF3D knockdown. Standard procedures were followed for cDNA synthesis, and RT-qPCR was executed using the Bio-Rad S1000 system with HieffTMqPCR SYBR^®^ Green Master Mix (Low Rox Plus; Yeasen, Shanghai, China). Supplementary Table 1 contains the information of the primers used. To standardize the concentration of each transcript, we normalised them to the GAPDH mRNA level using the 2^−ΔΔCT^ method [24]. Statistical comparisons were conducted using the paired Student’s *t*-test, facilitated by GraphPad Prism software (Version 8.0, San Diego, CA, USA).

### Western blot

FaDu cells were lysed in ice-cold Wash Buffer (1× PBS, 0.1% SDS, 0.5% NP-40, and 0.5% sodium deoxycholate), supplemented with a protease inhibitor cocktail (Roche, Basel, Switzerland), and incubated on ice for 30 minutes. Subsequently, samples were boiled for 10 minutes in boiling water with 1X SDS sample buffer and separated on 10% SDS-PAGE. Membranes were then subjected to incubation with primary antibodies: EIF3D antibody (1:1,000, 66024-1-Ig, Proteintech, China) and GAPDH (1:1,000, 60004-1-Ig, Proteintech), followed by exposure to HRP-conjugated secondary antibodies. Detection of the bound secondary antibody (anti-rabbit, 1:10,000, SA00001-2, Proteintech, China, or anti-mouse, 1:10,000, AS003, ABclonal, China) was carried out using enhanced chemiluminescence (ECL) reagent (170506, Bio-Rad, Hercules, CA, USA).

### Cell proliferation assay

We conducted the cell proliferation assay employing the Cell Counting kit-8 (CCK-8, 40203ES76, Yeasen, Shanghai, China). Briefly, FaDu cells were seeded at a density of 10,000 cells per well in 96-well culture plates. The cells in both the control and experimental groups received their respective treatments, while vials without cells served as blank controls. Following incubation for 0, 24, 48, and 72 hours at 37°C with 5% CO_2_, we introduced 10 μl of CCK-8 solution into the culture medium and incubated it for an additional 3 hours at 37°C. We measured the optical density of the cells at an absorbance of 450 nm using a Microplate Reader (ELX800, Biotek, USA). The cell proliferation rate was calculated using the formula: proliferation rate = (experimental OD value - blank OD value)/(control OD value - blank OD value) × 100%.

### Apoptosis assay

For the detection of cell apoptosis, we employed an Annexin V-APC/7-ADD apoptosis detection kit (KGA1026, KeyGEN BioTECH, China) following the manufacturer’s instructions. Specifically, FaDu cells were seeded into six-well plates and cultured for 24 hours before transfection with the plasmid for 48 hours. Subsequently, both treated and control cells were mixed with 5 μl Annexin V-APC and incubated at room temperature in the dark for 5 minutes, followed by incubation with 5 μl of 7-AAD reagents for another 5 minutes in the dark. The samples were then subjected to flow cytometry analysis using FACSCanto (BD, La Jolla, CA, USA).

### Invasion assay

*In vitro* invasion assays were performed using transwell chambers (3422, Corning, USA). These chambers featured an 8 μm filter precoated with a thin layer of Matrigel (356234, BD Biosciences, USA), diluted at a ratio of 1:8 using serum-free medium. A total of 100 μl of the diluted matrigel was incubated in the chambers for 1 hour at 37°C with 5% CO_2_, after which unsolidified supernatant was removed. Subsequently, 5 × 10^4^–10^5^ cells in 0.2 ml of serum-free medium were added to the inserts, while the transwell chambers were placed in medium containing 600 μl of 10% FBS (10091148, Gibco, China) as a chemoattractant in the lower chamber. The cells were then incubated for 24–48 hours at 37°C with 5% CO_2_. After the incubation period, cells remaining on the upper membrane surface of the inserts were removed using a cotton swab, and the total number of invading cells that had entered the lower chamber were fixed with 4% paraformaldehyde (P0099, Beyotime, China) for 20 minutes. These cells were subsequently stained with 0.1% crystal violet (C0121, Beyotime, China) and observed and counted under an inverted microscope (MF52-N, Mshot, China) at 200× magnification.

### Cell migration assay

*In vitro* migration assays were performed using transwell chambers (3422, Corning, USA). 5 × 10^4^–10^5^ cells in 0.2 ml serum-free medium were added to the transwell chambers with 8 μm filter, then the chambers inserted in medium with 600 ul 10% FBS (10091148, Gibco, China) served as a chemo-attractant in the lower chamber, and incubated for 24–48 h at 37°C and 5% CO_2_. Cells remaining on the upper membrane surface of the inserts were then removed with a cotton swab, and the total number of cells that migrated into the lower chamber were fixed by 4% paraformaldehyde (P0099, Beyotime, China) for 20 min, then stained with 0.1% crystal violet (C0121, Beyotime, China). The migration cells were observed and counted under inverted microscope (MF52-N, Mshot, China) at 200× magnification.

### RNA extraction and sequencing

Total RNA was treated with RQ1 DNase (Promega, Madison, WI, USA) to remove DNA. The quality and quantity of the purified RNA were determined by the absorbance at 260 nm/280 nm (A260/A280). RNA integrity was further verified by 1.5% agarose gel electrophoresis. For each sample, 1 μg of total RNA was used for RNA-seq library preparation. mRNAs were captured by VAHTS mRNA capture Beads (N401, Vazyme, China). The purified RNA was treated with RQ1 DNase (Promega) to remove DNA before used for directional VAHTS Universal V8 RNA-seq Library Prep Kit for Illumina (NR605) Polyadenylated mRNAs were purified and fragmented. Fragmented mRNAs were converted into double strand cDNA. Following end repair and A tailing, the DNAs were ligated to Adaptor (N323). After purification of ligation product and size fractioning to 300–500 bps, the ligated products were amplified and purified, quantified and stored at −80°C before sequencing. The strand marked with dUTP (the 2nd cDNA strand) is not amplified, allowing strand-specific sequencing. For high-throughput sequencing, the libraries were prepared following the manufacturer’s instructions and applied to Illumina Novaseq 6000 system for 150 nt paired-end sequencing.

### RNA-seq raw data cleaning and alignment

Raw reads containing more than 2-N bases were first discarded. Then adaptors and low-quality bases were trimmed from raw sequencing reads using FASTX-Toolkit (Version 0.0.13). The short reads less than 16 nt were also dropped. After that, clean reads were aligned to the GRCh38 genome by HISAT2 [25] allowing 4 mismatches. Uniquely mapped reads were used for gene reads number counting and FPKM calculation (fragments per kilobase of transcript per million fragments mapped) [26].

### Alternative splicing analysis

We defined and quantified alternative splicing events (ASEs) and regulated alternative splicing events (RASEs) between the samples using the ABLas pipeline, as previously described [27, 28]. In summary, ABLas detects ten types of ASEs based on splice junction reads. These include exon skipping (ES), alternative 5′ splice site (A5SS), alternative 3′ splice site (A3SS), mutually exclusive exons (MXE), mutually exclusive 5′ UTRs (5pMXE), mutually exclusive 3′ UTRs (3pMXE), cassette exon, A3SS&ES, and A5SS&ES.

To assess EIF3D-regulated ASEs, we conducted a Student’s *t*-test to evaluate the significance of the ratio alteration of ASEs. Events that reached significance at the *P*-value cutoff, corresponding to a false discovery rate cutoff of 5%, were considered EIF3D-regulated ASEs.

### Functional enrichment analysis

For sorting out functional categories of Differentially Expressed Genes (DEGs), we identified Gene Ontology (GO) terms and KEGG pathways using the KOBAS 2.0 server [29]. The hypergeometric test and the Benjamini-Hochberg FDR controlling procedure were employed to determine the enrichment of each term.

### Statistical analysis

Gene expression levels were represented as mean ± standard deviation and analyzed using R software (v4.1.3). The cut-off value for EIF3D expression was determined using the median method of gene expression. We employed a Student’s *t*-test to compare the two groups and utilized Kaplan-Meier curves with the log-rank test to assess overall survival in different groups. Furthermore, univariate and multivariate Cox regression analyses were conducted to evaluate the significance of EIF3D expression and clinicopathologic features for overall survival. Statistical significance was defined as a *P*-value < 0.05.

### Data availability

The data utilized in this study are accessible on the TCGA website, and for experimental data inquiries, please contact the corresponding author.

## RESULTS

### EIF3D expression is upregulated in HNSC and associated with poor prognosis

**Figure 1:**
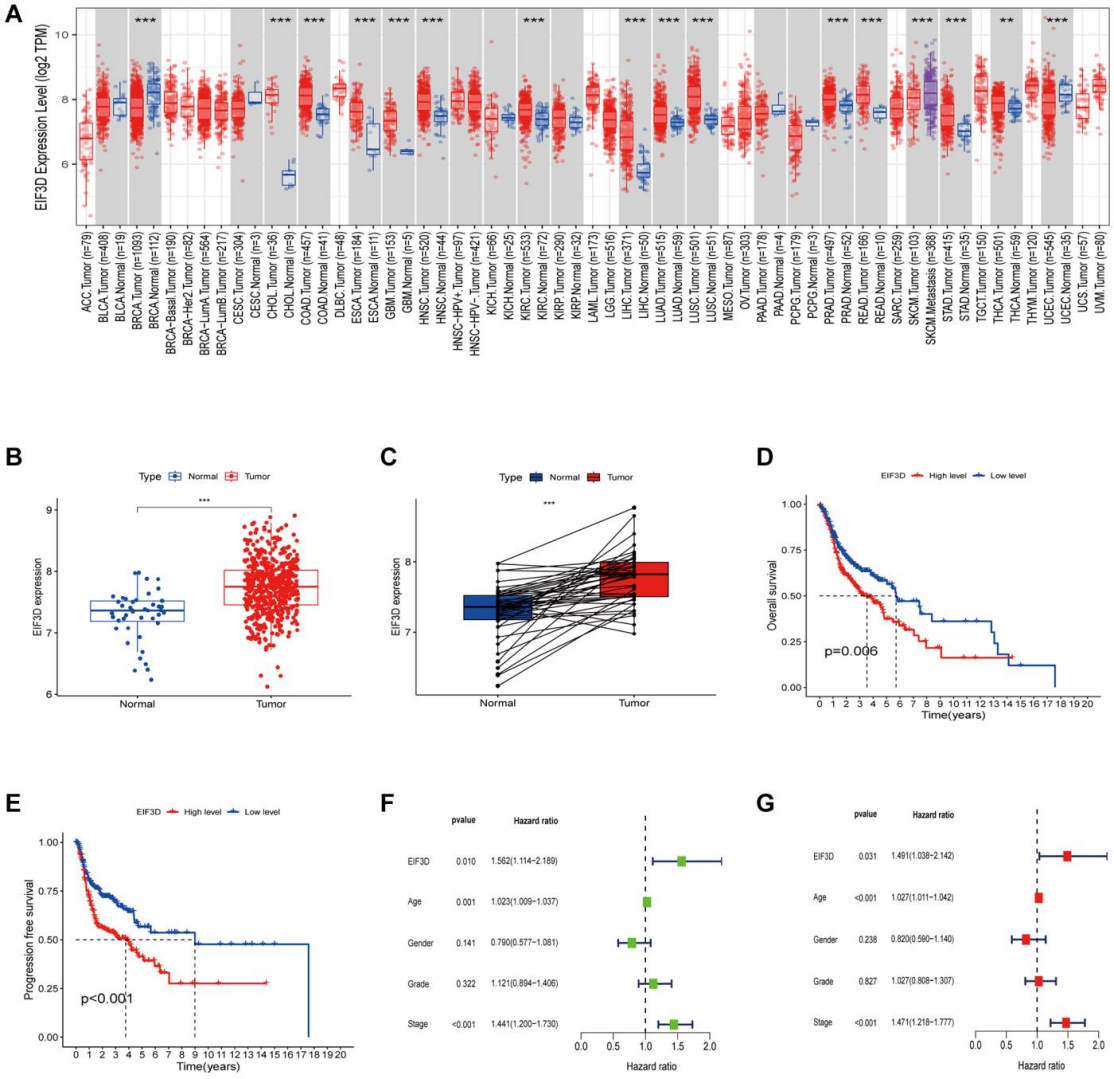
**Expression level and prognosis of EIF3D in HNSC.** (**A**) Box plot illustrating the expression level of EIF3D in pan-cancer dataset. (**B**, **C**) Box plots displaying EIF3D expression levels in a cohort of 566 HNSC samples retrieved from the TCGA database, encompassing 522 tumor samples and 44 normal samples. Error bars indicate the mean ± SEM. Significance denoted by *P* < 0.05. (**D**, **E**) Survival curves depicting the impact of EIF3D expression on HNSC patient survival, as determined from the TCGA databases. (**F**, **G**) Univariate and multivariate Cox regression analyses. Significance levels: ^*^P-value < 0.05, ^**^P-value < 0.01, ^***^P-value < 0.001.

In our pan-cancer analysis, significant differential expression of EIF3D was observed in breast cancer (BRCA), bile duct cancer (CHOL), colon and rectal cancer (COAD), and HNSC, with a noteworthy overexpression observed across most tumor types (*P* < 0.01, as depicted in Figure 1A). To delve deeper into the role of EIF3D in HNSC, we analysed its mRNA expression profile in 522 HNSC samples along with 44 normal samples from TCGA database. This analysis revealed a substantial upregulation of EIF3D in HNSC (*P* < 0.001, Figure 1B, 1C), and heightened EIF3D levels were linked to shorter overall survival (OS) (*P* = 0.006, Figure 1D) and progression-free survival (PFS) (*P* < 0.001, Figure 1E). Univariate Cox regression analysis (*P* = 0.010, HR = 1.562, 95% CI 1.114–2.189) demonstrated a significant association between high EIF3D expression and reduced OS in HNSC patients (Figure 1F). Multivariate Cox regression analysis further established EIF3D (*P* = 0.031, HR = 1.491, 95% CI 1.038–2.142) as an independent prognostic parameter for HNSC patients (Figure 1G). These findings underscore the upregulation of EIF3D in HNSC and its correlation with adverse prognosis in these patients.

### EIF3D has great potential in the regulation of tumor microenvironment

**Figure 2:**
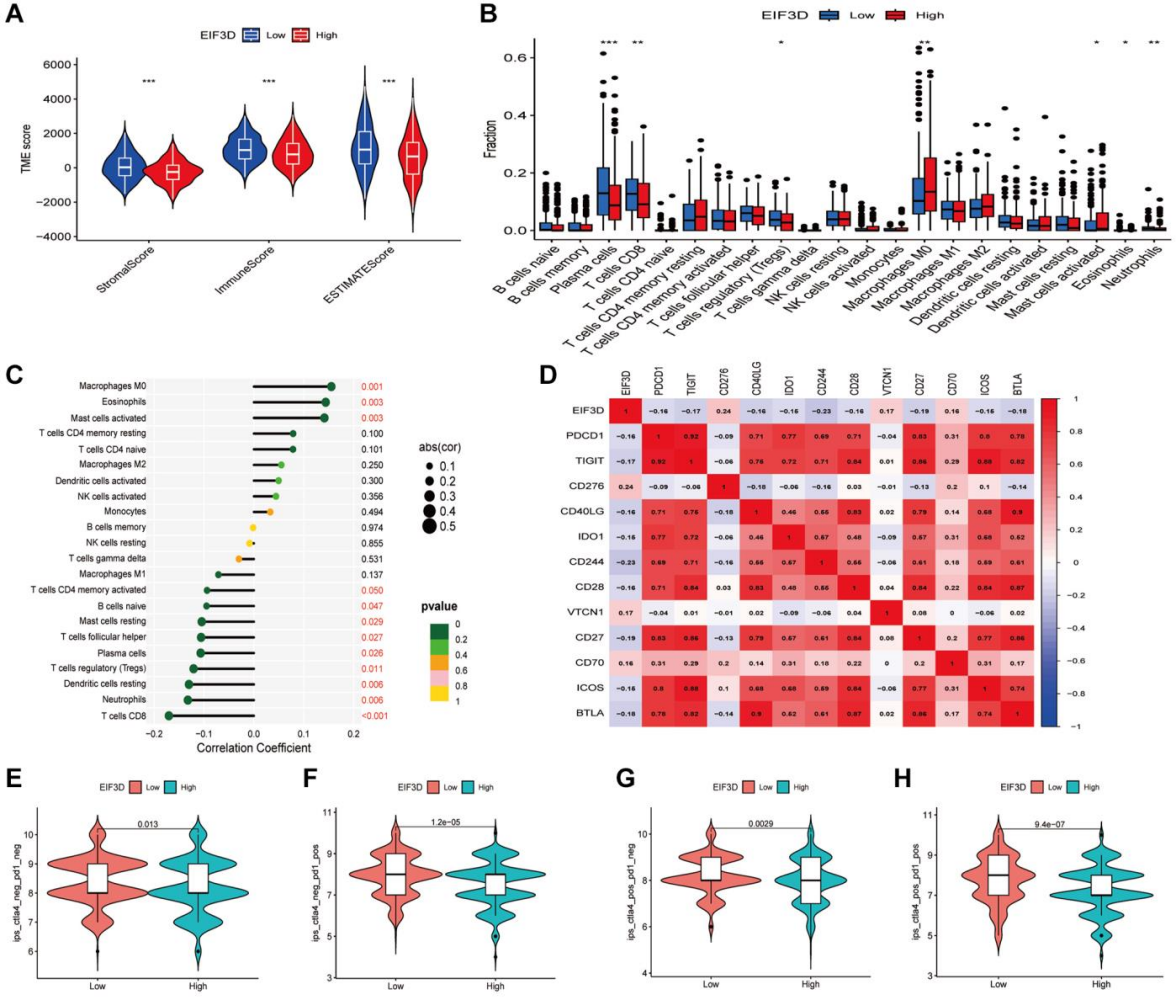
**Correlation between the EIF3D and the tumor microenvironment and immune checkpoint genes.** (**A**) Correlation analysis of ImmuneScore, StromalScore, and ESTIMATEScore with EIF3D. (**B**) Box plots illustrating variations in immune cell infiltration proportions between high-EIF3D and low-EIF3D patient groups. (**C**) Lollipop graph depicting the correlation between immune infiltration and EIF3D. (**D**) Correlation analysis between EIF3D and immune checkpoint genes. (**E**–**H**) Evaluation of immunotherapy outcomes involving anti-CTLA4 and anti-PD1 inhibitors in EIF3D high and low expression groups. Error bars denote mean ± SEM. Significance levels: ^*^P-value < 0.05, ^**^P-value < 0.01, ^***^P-value < 0.001.

Comparing patients with high EIF3D expression to those with low EIF3D expression, results revealed significantly elevated StromalScores, ImmuneScores, and ESTIMATEscores (*P* < 0.001, Figure 2A), indicating that EIF3D plays a pivotal role in regulating the TME. Employing the CIBERSORT algorithm, we analysed the proportions of 22 immune cell types in HNSC patients and presented the relative proportions in EIF3D high and low expression groups using box plots (Figure 2B). Among these, the EIF3D-low expression group exhibited higher proportions of plasma cells, CD8T cells, Tregs cells, and neutrophils compared to the EIF3D-high expression group. Conversely, the EIF3D-high expression group demonstrated a higher proportion of M0 macrophages, activated mast cells, and eosinophils. Correlation analysis revealed positive correlations between EIF3D and M0 macrophage infiltration, eosinophils, and activated mast cells, while negative correlations were observed with naive B cells, resting mast cells, follicular helper T cells, plasma cells, Tregs, resting dendritic cells, neutrophils, and CD8T cells (Figure 2C).

We further explored the correlation between EIF3D and immune checkpoint inhibitors using the expression levels of EIF3D and 47 immune checkpoint-associated genes. EIF3D exhibited significant associations with 13 immune checkpoint-related genes (*P* < 0.001). Notably, EIF3D expression displayed positive correlations with CD276, VTCN1, and CD70, while showing negative correlations with PD1, CD244, and TIGIT (Figure 2D). ImmuneScores were also calculated for anti-CTLA4 and anti-PD1 inhibitors, with higher ImmuneScores indicating enhanced treatment efficacy. Significant differences were observed in CTLA4-negative-PD1-positive, CTLA4-positive-PD1-negative, and CTLA4-positive-PD1-positive samples (Figure 2E–2H), indicating that anti-PD1, anti-CTLA4, and the combination of anti-PD1 and anti-CTLA4 therapies are more effective in patients with low EIF3D expression compared to those with high EIF3D expression.

### EIF3D affects the differentially spliced IGAS event

**Figure 3:**
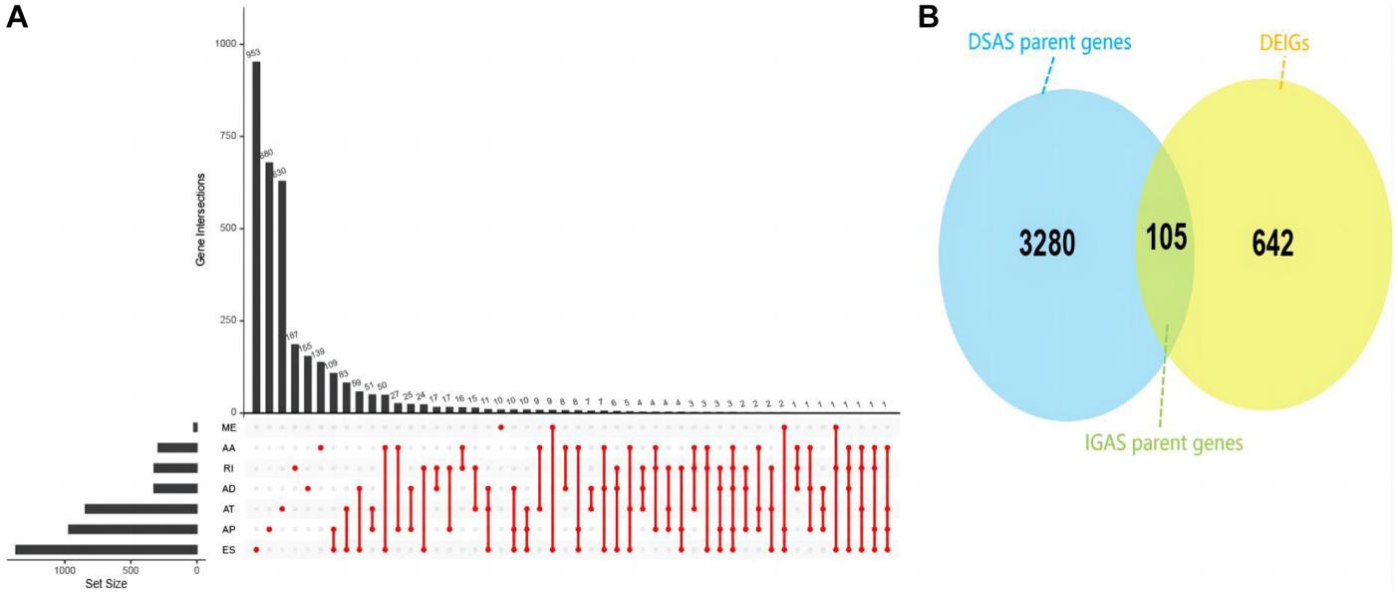
**Overview of IGAS events and parent genes in HNSC.** (**A**) UpSet plot illustrating interactions among the seven AS types of DSAS parent genes detected. (**B**) Venn plot displaying the intersection between DSAS parent genes and DEIGs.

In our analysis, we uncovered a total of 42,849 ASEs affecting 10,124 genes in HNSC patients. ES emerged as the most prevalent ASE type, followed by AT, AP, AA, AD, RI and ME (Supplementary Figure 1). Our investigation into DSAS between the EIF3D high and low expression groups unveiled 5,916 such events impacting 3,385 genes. Once again, ES constituted to be the majority of aberrantly regulated AS events (Figure 3A). Notably, in these groups, we identified 747 DEIGs through differential expression analysis, among them 105 DSAS parent genes belonged to the DEIGs category, which referred to as IGAS parent genes (Figure 3B). These findings strongly suggest that EIF3D may function as an RNA splicing regulator, influencing the prognosis of HNSC patients through its modulation of IGAS events.

### Knockdown of EIF3D inhibits cellular proliferation, migration and invasion, and promotes apoptosis in FaDu cells

**Figure 4:**
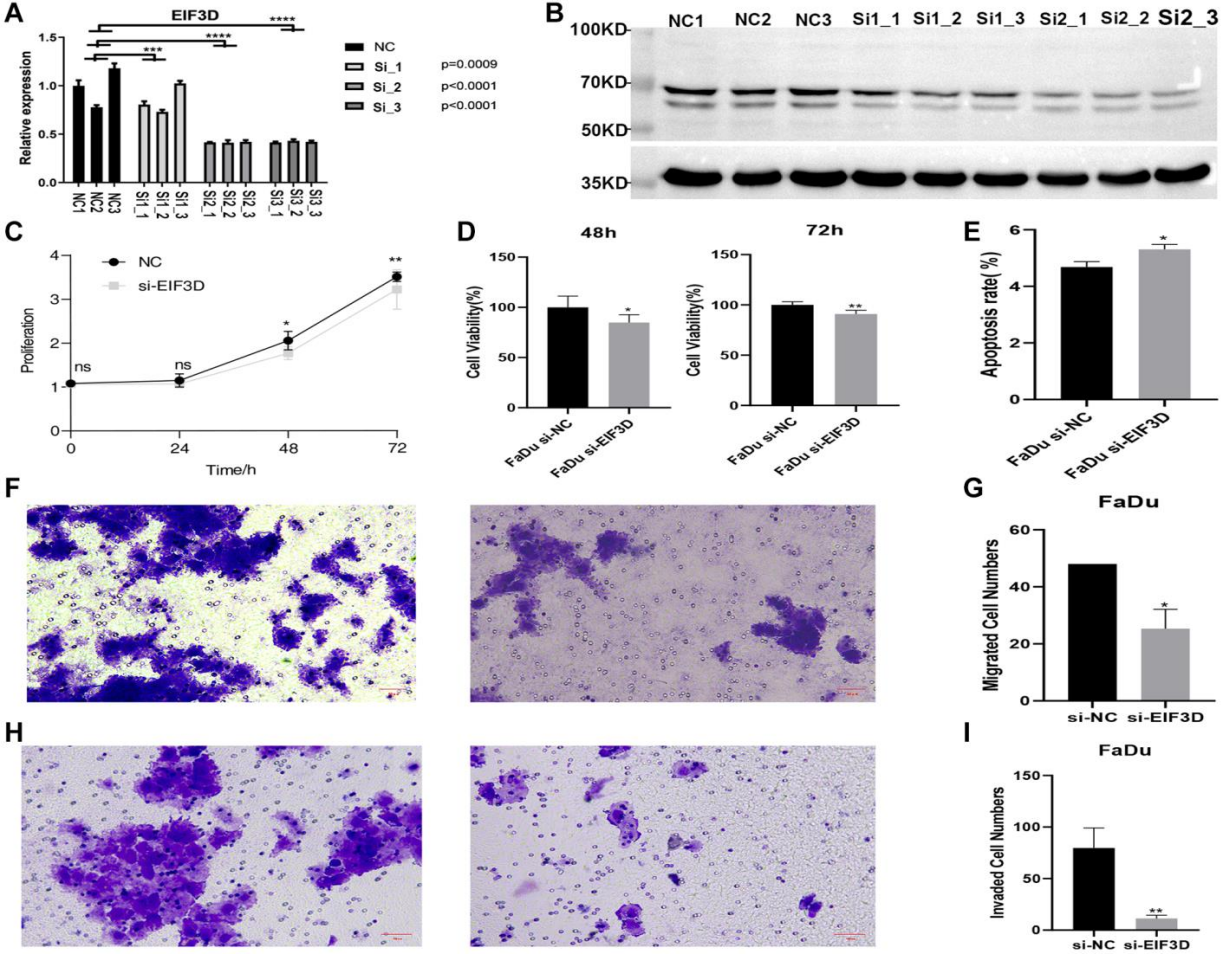
**Knockdown of EIF3D inhibits cellular proliferation and migration, and promotes apoptosis in FaDu cells.** (**A**) Bar plot depicting the RT-qPCR results for control and treatment samples (NC represents the control sample, and Si represents the experimental sample). (**B**) Western blot results. (**C**, **D**) Proliferation results of FaDu cells after EIF3D knockdown. (**E**) Apoptosis results of FaDu cells after EIF3D knockdown. (**F**, **G**) Cell migration results of FaDu cells after EIF3D knockdown. (**H**, **I**) Cell invasion results of FaDu cells after EIF3D knockdown. ^*^P-value < 0.05, ^**^P-value < 0.01, ^***^P-value < 0.001.

To discern the biological role of EIF3D in HNSC, this study established an EIF3D knockdown cell model using FaDu cell lines. The mRNA levels of EIF3D were notably lower in the EIF3D knockdown cell samples compared to the siRNA control (*P* < 0.001, Figure 4A). Similarly, in the cell model with reduced EIF3D expression, protein levels of EIF3D exhibited a significant decrease (Figure 4B). These results affirm the successful attenuation of EIF3D expression in FaDu cells. Subsequent analysis revealed that following EIF3D knockdown, the proliferation of FaDu cells was significantly curtailed at 48 and 72 hours (Figure 4C, 4D). Flow cytometry demonstrated that down-regulation of the EIF3D gene substantially increased the overall apoptosis rate of FaDu cells (Figure 4E). Furthermore, *in vitro* EIF3D downregulation significantly hampered the migration and invasion abilities of FaDu cells (Figure 4F–4I). Collectively, these results underscore the role of EIF3D in promoting FaDu cell proliferation, migration, and invasion while inhibiting apoptosis, providing further evidence that EIF3D plays a pro-cancer role in the progression of HNSC.

### EIF3D regulates immune-related gene expression in FaDu cells

**Figure 5:**
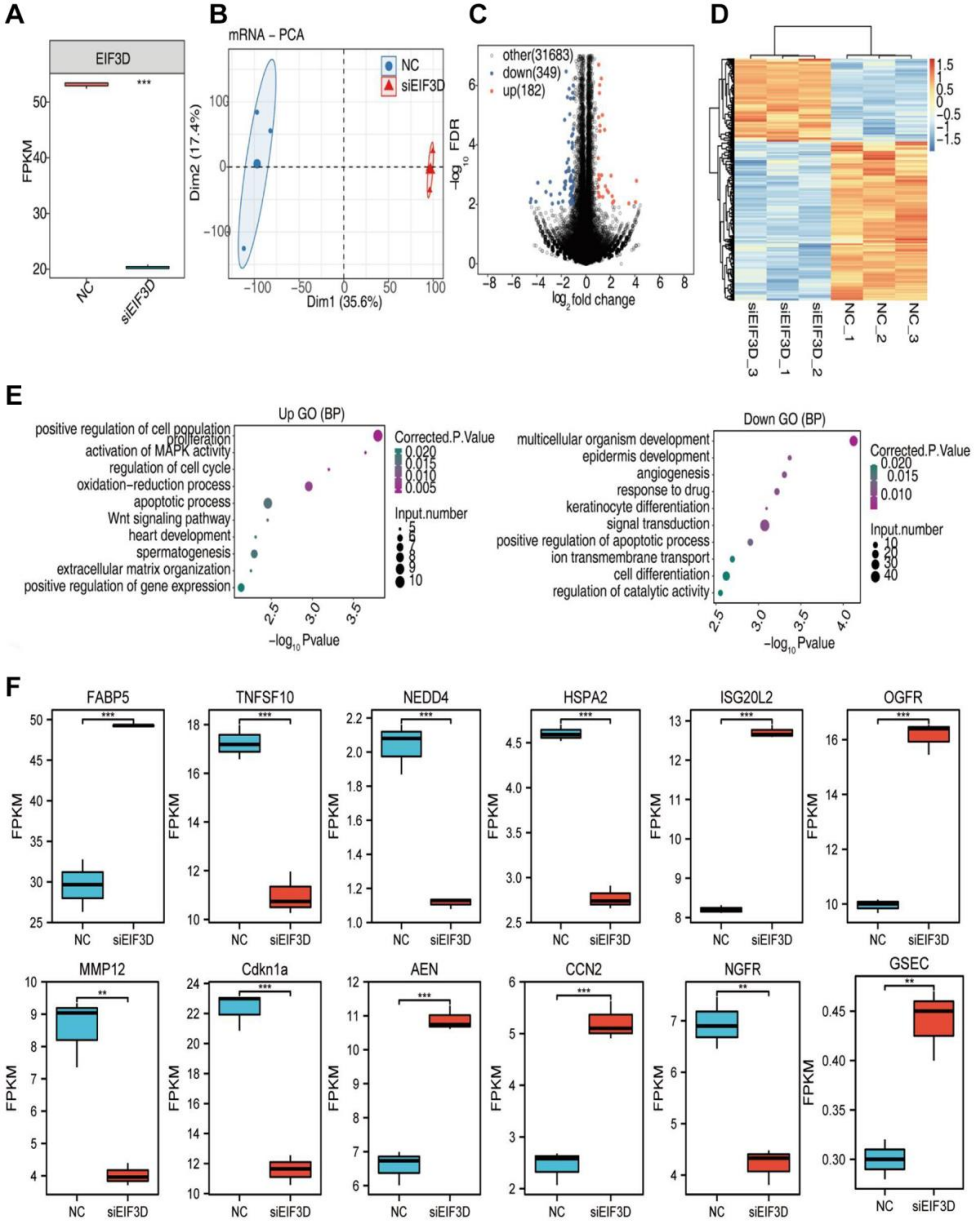
**EIF3D regulates gene expression in FaDu cells.** (**A**) Boxplot illustrating the expression levels of EIF3D in RNA-seq data. (**B**) PCA based on FPKM values of all detected genes. The ellipse for each group represents the confidence ellipse. (**C**) Volcano plot displaying all differentially expressed genes (DEGs) between treatment and control samples with DEseq2. *P*-value < 0.01 and FC (fold change) ≥ 1.5 or ≤ 0.67. (**D**) Hierarchical clustering heatmap depicting expression levels of all DEGs. (**E**) Bubble diagram presenting the most enriched GO biological process results of the up-regulated and down-regulated DEGs. (**F**) Boxplot showing the expression pattern and statistical differences of immune-related DEGs. Error bars represent mean ± SEM. ^*^P-value < 0.05, ^**^P-value < 0.01, ^***^P-value < 0.001.

Moreover, we explored the impact of down-regulating EIF3D on gene expression using RNA-seq (Figure 5A). The scatterplot displayed distinctive clustering patterns of the siEIF3D group and the normal control (NC) group after Principal Component Analysis (PCA) analysis (Figure 5B). Leveraging RNA-seq data, we uncovered potential gene targets influenced by EIF3D at the transcriptional level. Employing the R package “DESeq2” to screen for Differentially Expressed Genes (DEGs) between the two groups with the criteria (|log_2_FC| > 3/2 or ≤ 2/3 and *P* < 0.01), our analysis revealed that silencing the EIF3D gene in FaDu cells led to differential expression in 531 genes, comprising 182 up-regulated DEGs and 349 down-regulated DEGs (Figure 5C). Furthermore, the heatmap depicted the expression patterns of DEGs in the siEIF3D and NC groups (Figure 5D). Notably, over 65% of the genes among the 531 DEGs were down-regulated.

To gain insights into the potential biological roles of EIF3D, we conducted GO and KEGG enrichment analyses based on the 531 DEGs. Among the upregulated 182 DEGs, notable enrichments included processes such as positive regulation of cell population proliferation, activation of MAPK activity, regulation of the cell cycle, oxidation-reduction processes, apoptotic pathways, Wnt signaling, heart development, spermatogenesis, extracellular matrix organization, and positive regulation of gene expression. Conversely, the significantly down-regulated 349 DEGs were predominantly associated with multicellular organism development, epidermal development, angiogenesis, drug response, keratinocyte differentiation, signal transduction, positive regulation of apoptosis, ion transmembrane transport, cell differentiation, and regulation of catalytic activity, among other biological processes (Figure 5E). Moreover, we identified 52 immune-related genes among the 531 DEGs and selected 12 of these for further analysis. Within this subset, the expression of TNFSF10, NEDD4, HSPA2, MMP12, CDKN1A, and NGFR decreased in siEIF3D-treated FaDu cells, while FABP5, ISG20L2, OGFR, AEN, CCN2, and GSEC showed increased expression (Figure 5F). Remarkably, this study also showed a significant down-regulation of the alternative splicing factor NOVA2 among the DEGs. Taken together, these results imply that EIF3D may influence HNSC cell proliferation, apoptosis, angiogenesis, and cell cycle regulation by modulating the expression levels of immune-related genes and AS factors.

### EIF3D regulates IGAS event in FaDu cells

**Figure 6:**
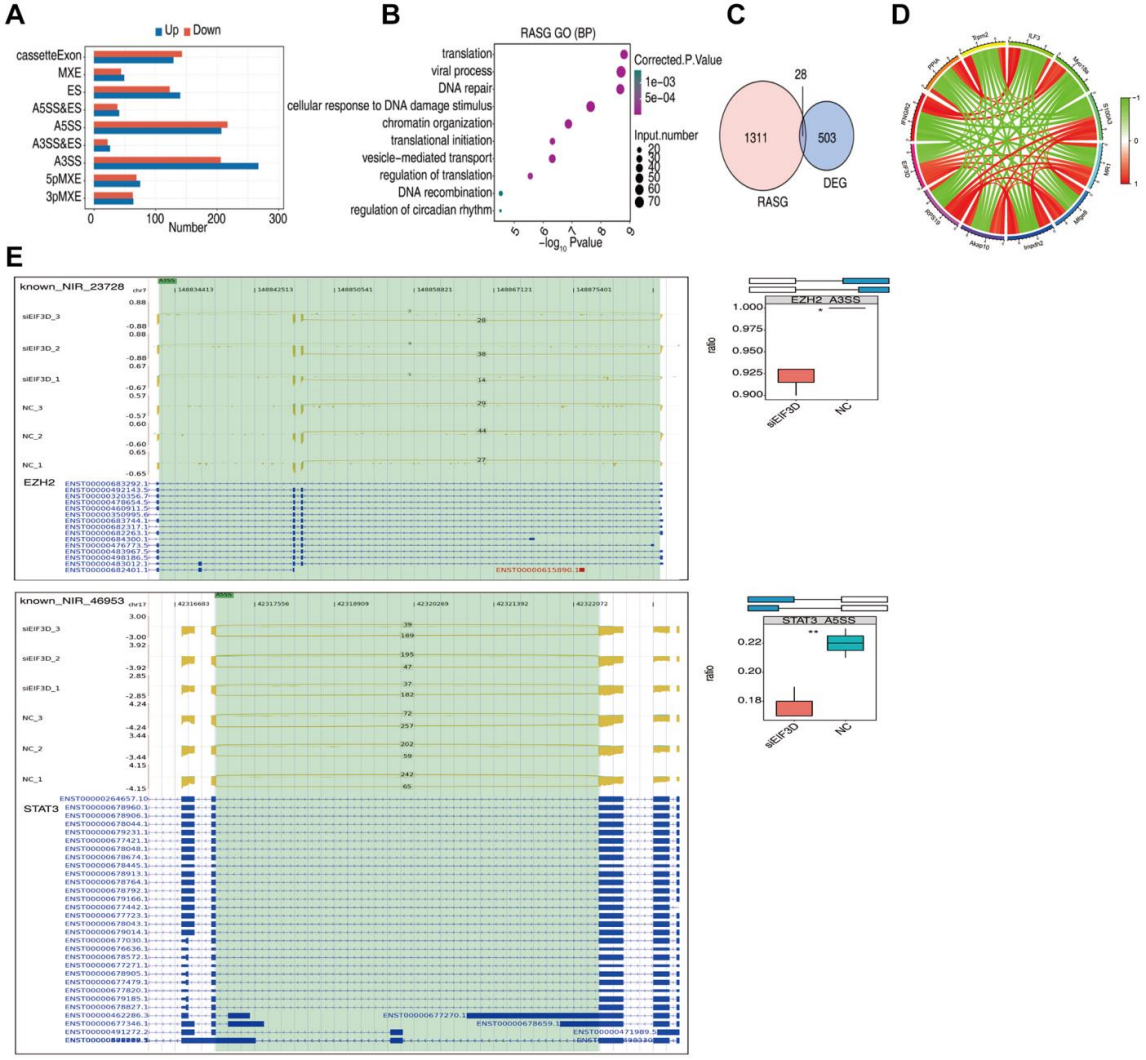
**EIF3D influences the alternative splicing of genes in FaDu cells.** (**A**) Bar plot displaying the number of all significantly regulated alternative splicing events (RASEs). The X-axis represents the RASE number, while the Y-axis represents the different types of AS events. "Up" indicates that the splicing pattern occurs in a higher proportion in the siEIF3D group than in the NC group, while "down" indicates the opposite. (**B**) Bubble diagram presenting the most enriched GO biological process results of RASGs. (**C**) Venn diagram illustrating the overlap in gene numbers between RASGs and DEGs. (**D**) Chord diagram demonstrating the co-expression of EIF3D with immune-associated RASGs. (**E**) Schematic diagrams depicting the structures of ASEs. RNA-seq validation of ASEs is displayed at the bottom of the right panel. Error bars represent mean ± SEM. ^*^P-value < 0.05, ^**^P-value < 0.01, ^***^P-value < 0.001.

In recent years, the close association between AS and the TME has garnered attention [21, 22]. To investigate the mechanistic role of EIF3D in HNSC, we conducted a comprehensive analysis of EIF3D-regulated ASEs. We detected a total of 114,901 ASEs, encompassing 53,373 known ASEs and 61,528 new ASEs. These AS events could be categorized into nine main types, including MXE, ES, Cassette Exon, A5SS&ES, A3SS&ES, A3SS, 5pMXE, 3pMXE (Figure 6A).

For the analysis of EIF3D-regulated ASEs, we employed a *t*-test to compare the changes in AS levels of genes with the same splice phenotype between two different samples, applying a significant difference ASE screening criterion of *p*-value ≤ 0.05. The results unveiled 998 DSAS events that were more prevalent in the siEIF3D group compared to the NC group, with higher proportions of 3pMXE, 5pMXE, A3SS, A3SS&ES, A5SS&ES, ES, and MXE events in the siEIF3D group.

Subsequently, we conducted GO enrichment analysis of EIF3D-regulated Differential Alternative Splicing Genes (RASG). The outcomes indicated that EIF3D-regulated ASEs are linked to various biological processes, including translation, viral processes, DNA repair, cellular response to DNA damage stimulus, chromatin organization, translation initiation, vesicle-mediated transport, translation regulation, DNA recombination, and regulation of circadian rhythm (Figure 6B).

Moreover, overlapping analysis of DEGs and RASGs identified 28 genes that were both DEGs and RASGs (Figure 6C). Additionally, we detected 129 IGAS events out of 80 immune-related genes in FaDu cells. Figure 6D illustrates that EIF3D exhibits significant co-expression with IGAS parent genes such as S100A3, RFXANK, PPIA, IFNGR2, ILF3, MDK, and RPS19, where red indicates positive correlation and green indicates negative correlation (|correlation coefficient| > 0.8, *P* < 0.05).

Within the list of ASEs, we selected events with significantly different ratios between treatment and control groups, leading to the identification of four IGAS events: EZH2, STAT3, TRPM2, and MFF. To validate EIF3D-regulated IGAS, we further investigated these four IGAS events using RNA-Seq techniques. EIF3D exhibited a downregulating effect on A3SS events, which potentially led to altered EZH2 expression. Additionally, EIF3D influenced the A5SS events of STAT3 (*P* < 0.05, Figure 6E). Furthermore, EIF3D modulated the 5pMXE events of MFF and the ES events of TRPM2 (*P* < 0.05, Supplementary Figures 2 and 3). These findings, consistent with the results of bioinformatics analysis, underscore EIF3D’s significant potential in regulating IGAS in the context of HNSC.

## DISCUSSION

In addition to its role in regulating gene expression levels, AS plays a pivotal role in ensuring the diversity of gene products. AS is a prevalent phenomenon, occurring in over 90% of mammalian genes, with particular prominence in the immune system [30]. Increasingly evident shows that AS exerts a profound influence on various tumorigenic processes, encompassing cancer initiation, progression, angiogenesis, and immune evasion [31]. However, comprehensive genome-wide analyses that could thoroughly characterize AS as a potential prognostic and predictive factor for HNSC patients are still lacking. In this study, we conducted a systematic analysis of EIF3D’s potential role through bioinformatics coupled with *in vitro* cell assays to elucidate its significance in HNSC. Our investigation encompassed prognostic capabilities, associations with clinical data, influence on the TME, variations in immune cell infiltration levels, sensitivity to immunotherapies, and regulation of IGAS events.

The translation of mRNAs in eukaryotes is meticulously regulated by multiple Eukaryotic Initiation Factors (EIFs), among which EIF3D has been identified to facilitate the translation of specific mRNAs independently of EIF4E12. Previous studies have demonstrated that the downregulation of EIF3D inhibits the growth of non-small cell lung cancer [32], breast cancer [33], and kidney cancer. Similarly, in our investigation, EIF3D exhibited significant overexpression in HNSC and displayed a negative correlation with OS and PFS. Mounting evidence suggests that dysfunction of immune cells within the HNSC TME contributes to immunosuppression, thereby influencing the disease’s progression and treatment outcomes [34, 35]. Dong et al. reported that cancer-associated fibroblasts (CAFs) within HNSC could suppress immune responses within the TME. Notably, CAFs exhibited a negative correlation with immune cell infiltration levels, prognosis, and treatment outcomes [36]. Our study extends this analysis by exploring the impact of EIF3D expression levels on the TME, immune cell infiltration, and immune checkpoint genes, shedding light on the potential role of EIF3D in these intricate processes. ImmunoScore, StromalScore, and ESTIMATEScore exhibited significant disparities between the high and low EIF3D expression groups, with all three scores being notably higher in the low EIF3D expression group. Furthermore, immune cell infiltration levels were elevated in the EIF3D low expression group, implying a potential correlation between a superior prognosis in patients with low EIF3D expression and heightened immune infiltration. Specifically, the EIF3D low expression group exhibited higher proportions of plasma cells, CD8+ T cells, and Tregs among HNSC patients. Prior investigations have established a positive relationship between increased CD8+ T cell infiltration within head and neck tumor tissue and improved survival rates [37], a trend also observed in other malignancies such as pancreatic cancer [38] and breast cancer [39]. Tregs play a pivotal role in preserving self-tolerance and immune equilibrium [40].

Furthermore, our analysis unveiled a positive correlation between EIF3D with CD276, VTCN1, and CD70, while displaying a negative correlation with PDCD1, CD244, and TIGIT. Prior studies had highlighted the AS of PDCD1 [41], particularly in the context of exon 3, which generates a PD-1Δ3 protein isoform capable of antagonizing PD-1 [42]. However, activation of the PD-1 signaling pathway can foster a TME conducive to tumor growth [43]. Additionally, research has indicated that differential splicing products of CD244 in mice, namely m2B4-L and m2B4-S, which can influence the infiltration of NK cells. Our focused analysis on EIF3D-regulated AS events, particularly Immune-Related Gene Alternative Splicing (IGAS), highlighted significant differential expression involving 105 IGAS parent genes. These findings align with prior research indicating extensive AS of pre-mRNAs in human immune-related genes [44]. Collectively, these results suggest that EIF3D may reshape the TME through AS modulation, thereby influencing patient prognosis and responsiveness to immunotherapeutic interventions.

This study delved further into the potential functions of EIF3D through *in vitro* experiments. The knockdown of EIF3D was observed to enhance apoptosis, impeding the proliferation, migration, and invasion of FaDu cells. Downregulation of EIF3D resulted in differential expression of 531 genes, 52 of which were associated with immune responses. Notably, immune-related genes such as TNFSF10, NEDD4, HSPA2, MMP12, CDKN1A, and NGFR exhibited reduced expression, while FABP5, ISG20L2, OGFR, AEN, CCN2, and GSEC showed increased expression. For instance, TNFSF10-deficiency in breast tumors has been linked to decreased tumor-infiltrating CD4+ and CD8+ T cells in a mouse model of breast cancer [45]. High NEDD4 expression in melanoma tissue is associated with poor patient prognosis and promotes tumor progression by inhibiting T-cell-mediated killing of melanoma cells [46]. MMP12 has been implicated in increasing FOXP3 Treg infiltration into tumor tissues, thereby enhancing tumor proliferation and immune evasion in HCC [47]. Emerging evidence also suggests that FABP5 expression in T cells may regulate lipid metabolism and T-cell function [48]. In melanoma patients, NGFR was found to be associated with immune rejection and has been shown to predict resistance to anti-PD-1 therapy, while pharmacological inhibition of NGFR can restore tumor sensitivity to T-cell attacks [49].

Furthermore, the downregulation of EIF3D led to significant upregulation of 182 genes, which primarily associated with the regulation of the cell cycle, apoptotic processes, and the Wnt signaling pathway. Conversely, 349 downregulated genes were linked to angiogenesis, positive regulation of apoptotic processes, and other pathways. Apoptosis is an integral part of the immune system, aiding in the maintenance of immune system homeostasis by inducing the death of T and B cells at specific time points to limit the immune response [50]. Notably, HNSC has been shown to evade immune recognition through constitutive resistance to Fas receptor (Fas)-mediated apoptosis [51]. Collectively, these findings suggest that EIF3D-mediated expression levels of immune-related genes and immune cell apoptosis may also be correlated with tumor responsiveness to immunotherapy.

Missplicing is a prevalent phenomenon in human cancers, with underutilized splice sites being particularly prone to mutagenesis [52, 53]. Genes exhibiting higher levels of AS tend to engage in more protein-protein interactions, thereby contributing to increased protein diversity. Additionally, splicing dysregulation can lead to isoform switching in oncogenes, enabling cancer cells to develop resistance to anticancer therapies [54]. In our study, we observed that the knockdown of EIF3D lead to alterations in various types of ASEs in FaDu cells. Moreover, RASGs are predominantly implicated in essential biological processes, including translation, viral processes, DNA repair, translation initiation, vesicle-mediated translocation, translation regulation, and DNA recombination. Furthermore, our RNA-seq analysis confirmed differences in alternative splicing for four immune-related genes (EZH2, STAT3, TRPM2, MFF). EZH2 plays a significant role in the regulation of immune cells, influencing their proliferation, differentiation, activation, and phenotype transformation [55]. EZH2-X9, a novel variant generated through alternative splicing in the mammalian central nervous system, acts to inhibit cell cycle progression and the expression of autophagy-related genes [56]. STAT3, which contains a tandem splice site, can produce specific isoforms, STAT3α and STAT3β with distinct cellular roles of tumor promoters and tumor suppressors [57]. Previous research has identified TRPM2-S as an alternative splicing product of TRPM2 that plays a crucial role in regulating channel activity and oxidative stress-induced cell death [58]. The MFF gene encodes nine splice variants, differing in the presence or absence of exon 1 and various combinations of exons 5, 6, and 7, which encode the core region of the protein [59]. Therefore, our findings suggest that EIF3D may function as an RNA splicing regulator, modulating immune-related gene alternative splicing events (IGAS) in HNSC.

Nevertheless, it is important to acknowledge certain limitations of this study. Firstly, our findings are primarily based on an EIF3D knockout cell model, and their clinical relevance should be further substantiated through subsequent clinical investigations. Secondly, while EIF3D is identified as a contributing factor in HNSC progression, we recognized that other molecular mechanisms may also play crucial roles in comprehending the full pathogenesis of HNSC. For instance, investigating the interactions between EIF3D and splicing factors warrants consideration.

## CONCLUSION

This study provides the first evidence that EIF3D can function as an RNA splicing regulator, orchestrating IGAS and promoting pro-cancer effects in HNSC by associating with immune-related transcripts. Furthermore, EIF3D appears to reshape the TME through its regulation of IGAS, subsequently influencing how HNSC responds to immunotherapies. Consequently, strategies aimed at reducing the expression of oncogenic isoforms and correcting aberrant alternative splicing events hold promise for enhancing the effectiveness of immunotherapy in HNSC.

## Supplementary Materials

Supplementary Figures

Supplementary Table 1

## References

[1] Mody MD, Rocco JW, Yom SS, Haddad RI, Saba NF. Head and neck cancer. Lancet. 2021; 398:2289–99. 10.1016/S0140-6736(21)01550-6 34562395

[2] Johnson DE, Burtness B, Leemans CR, Lui VWY, Bauman JE, Grandis JR. Head and neck squamous cell carcinoma. Nat Rev Dis Primers. 2020; 6:92. 10.1038/s41572-020-00224-3 33243986 PMC7944998

[3] Spilka R, Ernst C, Mehta AK, Haybaeck J. Eukaryotic translation initiation factors in cancer development and progression. Cancer Lett. 2013; 340:9–21. 10.1016/j.canlet.2013.06.019 23830805

[4] Zhang F, Xiang S, Cao Y, Li M, Ma Q, Liang H, Li H, Ye Y, Zhang Y, Jiang L, Hu Y, Zhou J, Wang X, et al. EIF3D promotes gallbladder cancer development by stabilizing GRK2 kinase and activating PI3K-AKT signaling pathway. Cell Death Dis. 2017; 8:e2868. 10.1038/cddis.2017.263 28594409 PMC5520919

[5] He J, Wang X, Cai J, Wang W, Qin X. High expression of eIF3d is associated with poor prognosis in patients with gastric cancer. Cancer Manag Res. 2017; 9:539–44. 10.2147/CMAR.S142324 29123423 PMC5661832

[6] Wang D, Jia Y, Zheng W, Li C, Cui W. Overexpression of *eIF3D* in Lung Adenocarcinoma Is a New Independent Prognostic Marker of Poor Survival. Dis Markers. 2019; 2019:6019637. 10.1155/2019/6019637 31885740 PMC6925810

[7] Maekawa M, Hiyoshi H, Nakayama J, Kido K, Sawasaki T, Semba K, Kubota E, Joh T, Higashiyama S. Cullin-3/KCTD10 complex is essential for K27-polyubiquitination of EIF3D in human hepatocellular carcinoma HepG2 cells. Biochem Biophys Res Commun. 2019; 516:1116–22. 10.1016/j.bbrc.2019.07.010 31280863

[8] Jiang M, Lu Y, Duan D, Wang H, Man G, Kang C, Abulimiti K, Li Y. Systematic Investigation of mRNA *N*^6^-Methyladenosine Machinery in Primary Prostate Cancer. Dis Markers. 2020; 2020:8833438. 10.1155/2020/8833438 33273988 PMC7676945

[9] Lee AS, Kranzusch PJ, Doudna JA, Cate JH. eIF3d is an mRNA cap-binding protein that is required for specialized translation initiation. Nature. 2016; 536:96–9. 10.1038/nature18954 27462815 PMC5003174

[10] Zhang W, Sun Y, Bai L, Zhi L, Yang Y, Zhao Q, Chen C, Qi Y, Gao W, He W, Wang L, Chen D, Fan S, et al. RBMS1 regulates lung cancer ferroptosis through translational control of SLC7A11. J Clin Invest. 2021; 131:e152067. 10.1172/JCI152067 34609966 PMC8592553

[11] Cieśla M, Ngoc PCT, Cordero E, Martinez ÁS, Morsing M, Muthukumar S, Beneventi G, Madej M, Munita R, Jönsson T, Lövgren K, Ebbesson A, Nodin B, et al. Oncogenic translation directs spliceosome dynamics revealing an integral role for SF3A3 in breast cancer. Mol Cell. 2021; 81:1453–68.e12. 10.1016/j.molcel.2021.01.034 33662273

[12] de la Parra C, Ernlund A, Alard A, Ruggles K, Ueberheide B, Schneider RJ. A widespread alternate form of cap-dependent mRNA translation initiation. Nat Commun. 2018; 9:3068. 10.1038/s41467-018-05539-0 30076308 PMC6076257

[13] Brito Querido J, Sokabe M, Kraatz S, Gordiyenko Y, Skehel JM, Fraser CS, Ramakrishnan V. Structure of a human 48*S* translational initiation complex. Science. 2020; 369:1220–7. 10.1126/science.aba4904 32883864 PMC7116333

[14] Lamper AM, Fleming RH, Ladd KM, Lee ASY. A phosphorylation-regulated eIF3d translation switch mediates cellular adaptation to metabolic stress. Science. 2020; 370:853–6. 10.1126/science.abb0993 33184215

[15] Thompson L, Depledge DP, Burgess HM, Mohr I. An eIF3d-dependent switch regulates HCMV replication by remodeling the infected cell translation landscape to mimic chronic ER stress. Cell Rep. 2022; 39:110767. 10.1016/j.celrep.2022.110767 35508137 PMC9127984

[16] Lee AS, Kranzusch PJ, Cate JH. eIF3 targets cell-proliferation messenger RNAs for translational activation or repression. Nature. 2015; 522:111–4. 10.1038/nature14267 25849773 PMC4603833

[17] Baralle FE, Giudice J. Alternative splicing as a regulator of development and tissue identity. Nat Rev Mol Cell Biol. 2017; 18:437–51. 10.1038/nrm.2017.27 28488700 PMC6839889

[18] Ruffin AT, Li H, Vujanovic L, Zandberg DP, Ferris RL, Bruno TC. Improving head and neck cancer therapies by immunomodulation of the tumour microenvironment. Nat Rev Cancer. 2023; 23:173–88. 10.1038/s41568-022-00531-9 36456755 PMC9992112

[19] Evrard D, Dumont C, Gatineau M, Delord JP, Fayette J, Dreyer C, Tijeras-Raballand A, de Gramont A, Delattre JF, Granier M, Aissat N, Garcia-Larnicol ML, Slimane K, et al. Targeting the Tumor Microenvironment through mTOR Inhibition and Chemotherapy as Induction Therapy for Locally Advanced Head and Neck Squamous Cell Carcinoma: The CAPRA Study. Cancers (Basel). 2022; 14:4509. 10.3390/cancers14184509 36139669 PMC9496893

[20] Laplagne C, Domagala M, Le Naour A, Quemerais C, Hamel D, Fournié JJ, Couderc B, Bousquet C, Ferrand A, Poupot M. Latest Advances in Targeting the Tumor Microenvironment for Tumor Suppression. Int J Mol Sci. 2019; 20:4719. 10.3390/ijms20194719 31547627 PMC6801830

[21] Zhong W, Wu Y, Zhu M, Zhong H, Huang C, Lin Y, Huang J. Alternative splicing and alternative polyadenylation define tumor immune microenvironment and pharmacogenomic landscape in clear cell renal carcinoma. Mol Ther Nucleic Acids. 2022; 27:927–46. 10.1016/j.omtn.2022.01.014 35211354 PMC8829526

[22] Liu J, Wang Y, Zhao X, Wang K, Wang C, Du J. Prognostic alternative splicing events related splicing factors define the tumor microenvironment and pharmacogenomic landscape in lung adenocarcinoma. Aging (Albany NY). 2022; 14:6689–715. 10.18632/aging.204244 36006412 PMC9467413

[23] Bhattacharya S, Andorf S, Gomes L, Dunn P, Schaefer H, Pontius J, Berger P, Desborough V, Smith T, Campbell J, Thomson E, Monteiro R, Guimaraes P, et al. ImmPort: disseminating data to the public for the future of immunology. Immunol Res. 2014; 58:234–9. 10.1007/s12026-014-8516-1 24791905

[24] Livak KJ, Schmittgen TD. Analysis of relative gene expression data using real-time quantitative PCR and the 2(-Delta Delta C(T)) Method. Methods. 2001; 25:402–8. 10.1006/meth.2001.1262 11846609

[25] Kim D, Langmead B, Salzberg SL. HISAT: a fast spliced aligner with low memory requirements. Nat Methods. 2015; 12:357–60. 10.1038/nmeth.3317 25751142 PMC4655817

[26] Trapnell C, Williams BA, Pertea G, Mortazavi A, Kwan G, van Baren MJ, Salzberg SL, Wold BJ, Pachter L. Transcript assembly and quantification by RNA-Seq reveals unannotated transcripts and isoform switching during cell differentiation. Nat Biotechnol. 2010; 28:511–5. 10.1038/nbt.1621 20436464 PMC3146043

[27] Jin L, Li G, Yu D, Huang W, Cheng C, Liao S, Wu Q, Zhang Y. Transcriptome analysis reveals the complexity of alternative splicing regulation in the fungus Verticillium dahliae. BMC Genomics. 2017; 18:130. 10.1186/s12864-017-3507-y 28166730 PMC5294800

[28] Xia H, Chen D, Wu Q, Wu G, Zhou Y, Zhang Y, Zhang L. CELF1 preferentially binds to exon-intron boundary and regulates alternative splicing in HeLa cells. Biochim Biophys Acta Gene Regul Mech. 2017; 1860:911–21. 10.1016/j.bbagrm.2017.07.004 28733224

[29] Xie C, Mao X, Huang J, Ding Y, Wu J, Dong S, Kong L, Gao G, Li CY, Wei L. KOBAS 2.0: a web server for annotation and identification of enriched pathways and diseases. Nucleic Acids Res. 2011; 39:W316–22. 10.1093/nar/gkr483 21715386 PMC3125809

[30] Joyce JA, Pollard JW. Microenvironmental regulation of metastasis. Nat Rev Cancer. 2009; 9:239–52. 10.1038/nrc2618 19279573 PMC3251309

[31] Misquitta-Ali CM, Cheng E, O'Hanlon D, Liu N, McGlade CJ, Tsao MS, Blencowe BJ. Global profiling and molecular characterization of alternative splicing events misregulated in lung cancer. Mol Cell Biol. 2011; 31:138–50. 10.1128/MCB.00709-10 21041478 PMC3019846

[32] Lin Z, Xiong L, Lin Q. Expression of Concern: Knockdown of eIF3d inhibits cell proliferation through G2/M phase arrest in non-small cell lung cancer. Med Oncol. 2018; 35:130. 10.1007/s12032-018-1178-4 30121714

[33] Fan Y, Guo Y. Knockdown of eIF3D inhibits breast cancer cell proliferation and invasion through suppressing the Wnt/β-catenin signaling pathway. Int J Clin Exp Pathol. 2015; 8:10420–7. 26617750 PMC4637565

[34] Zhang X, Shi M, Chen T, Zhang B. Characterization of the Immune Cell Infiltration Landscape in Head and Neck Squamous Cell Carcinoma to Aid Immunotherapy. Mol Ther Nucleic Acids. 2020; 22:298–309. 10.1016/j.omtn.2020.08.030 33230435 PMC7522342

[35] Huo M, Zhang Y, Chen Z, Zhang S, Bao Y, Li T. Tumor microenvironment characterization in head and neck cancer identifies prognostic and immunotherapeutically relevant gene signatures. Sci Rep. 2020; 10:11163. 10.1038/s41598-020-68074-3 32636465 PMC7341839

[36] Dong L, Sun Q, Song F, Song X, Lu C, Li Y, Song X. Identification and verification of eight cancer-associated fibroblasts related genes as a prognostic signature for head and neck squamous cell carcinoma. Heliyon. 2023; 9:e14003. 10.1016/j.heliyon.2023.e14003 36938461 PMC10018481

[37] Zhou C, Shen Y, Jin Y, Shen Z, Ye D, Shen Y, Deng H. A novel Pyroptosis-related long non-coding RNA signature for predicting the prognosis and immune landscape of head and neck squamous cell carcinoma. Cancer Med. 2022; 11:5097–112. 10.1002/cam4.4819 35567376 PMC9761069

[38] Nadella S, Burks J, Al-Sabban A, Inyang G, Wang J, Tucker RD, Zamanis ME, Bukowski W, Shivapurkar N, Smith JP. Dietary fat stimulates pancreatic cancer growth and promotes fibrosis of the tumor microenvironment through the cholecystokinin receptor. Am J Physiol Gastrointest Liver Physiol. 2018; 315:G699–712. 10.1152/ajpgi.00123.2018 29927319 PMC6293257

[39] Martín-Manzo MV, Lara C, Vargas-de-Leon C, Carrero J, Queipo G, Fonseca-Sanchez M, Mejia-Dominguez NR, Kershenobich D, Mummidi S, Zentella-Dehesa A, Hernandez J. Interaction of Breast Cancer and Insulin Resistance on PD1 and TIM3 Expression in Peripheral Blood CD8 T Cells. Pathol Oncol Res. 2019; 25:1233–43. 10.1007/s12253-019-00610-7 30759303

[40] Gedaly R, Cornea V, Turcios L, Edmisson JS, Harris DD, Watt DS, Chapelin F, Khurana A, Mei X, Liu C, Taylor I, Gonzalez-Valdivieso J, Mitchel H, et al. Anti-neoplastic sulfonamides alter the metabolic homeostasis and disrupt the suppressor activity of regulatory T cells. Sci Rep. 2022; 12:19112. 10.1038/s41598-022-23601-2 36352020 PMC9646802

[41] Nielsen C, Ohm-Laursen L, Barington T, Husby S, Lillevang ST. Alternative splice variants of the human PD-1 gene. Cell Immunol. 2005; 235:109–16. 10.1016/j.cellimm.2005.07.007 16171790

[42] Sorensen SF, Demuth C, Weber B, Sorensen BS, Meldgaard P. Increase in soluble PD-1 is associated with prolonged survival in patients with advanced EGFR-mutated non-small cell lung cancer treated with erlotinib. Lung Cancer. 2016; 100:77–84. 10.1016/j.lungcan.2016.08.001 27597284

[43] Sun J, Bai J, Jiang T, Gao Y, Hua Y. Modulation of *PDCD1* exon 3 splicing. RNA Biol. 2019; 16:1794–805. 10.1080/15476286.2019.1659080 31441370 PMC6844568

[44] Lynch KW. Consequences of regulated pre-mRNA splicing in the immune system. Nat Rev Immunol. 2004; 4:931–40. 10.1038/nri1497 15573128

[45] Han YJ, Zhang J, Hardeman A, Liu M, Karginova O, Romero R, Khramtsova GF, Zheng Y, Huo D, Olopade OI. An enhancer variant associated with breast cancer susceptibility in Black women regulates TNFSF10 expression and antitumor immunity in triple-negative breast cancer. Hum Mol Genet. 2023; 32:139–50. 10.1093/hmg/ddac168 35930348 PMC9837834

[46] Guo Y, Yang L, Lei S, Tan W, Long J. NEDD4 Negatively Regulates GITR via Ubiquitination in Immune Microenvironment of Melanoma. Onco Targets Ther. 2019; 12:10629–37. 10.2147/OTT.S212317 31824170 PMC6900405

[47] He MK, Le Y, Zhang YF, Ouyang HY, Jian PE, Yu ZS, Wang LJ, Shi M. Matrix metalloproteinase 12 expression is associated with tumor FOXP3^+^ regulatory T cell infiltration and poor prognosis in hepatocellular carcinoma. Oncol Lett. 2018; 16:475–82. 10.3892/ol.2018.8642 29928435 PMC6006382

[48] Jin R, Hao J, Yu J, Wang P, Sauter ER, Li B. Role of FABP5 in T Cell Lipid Metabolism and Function in the Tumor Microenvironment. Cancers (Basel). 2023; 15:657. 10.3390/cancers15030657 36765614 PMC9913835

[49] Boshuizen J, Vredevoogd DW, Krijgsman O, Ligtenberg MA, Blankenstein S, de Bruijn B, Frederick DT, Kenski JCN, Parren M, Brüggemann M, Madu MF, Rozeman EA, Song JY, et al. Reversal of pre-existing NGFR-driven tumor and immune therapy resistance. Nat Commun. 2020; 11:3946. 10.1038/s41467-020-17739-8 32770055 PMC7414147

[50] Ren J, Zhang X, Zhang Z, Pan J, Hao Z, Li J, Liu J. Apoptosis inhibition enhances induced pluripotent stem cell generation during T cell reprogramming. Biochem Biophys Res Commun. 2023; 656:30–7. 10.1016/j.bbrc.2023.03.024 36947964

[51] Xiao R, Allen CT, Tran L, Patel P, Park SJ, Chen Z, Van Waes C, Schmitt NC. Antagonist of cIAP1/2 and XIAP enhances anti-tumor immunity when combined with radiation and PD-1 blockade in a syngeneic model of head and neck cancer. Oncoimmunology. 2018; 7:e1471440. 10.1080/2162402X.2018.1471440 30393585 PMC6209421

[52] Rekad Z, Izzi V, Lamba R, Ciais D, Van Obberghen-Schilling E. The alternative matrisome: Alternative splicing of ECM proteins in development, homeostasis and tumor progression. Matrix Biol. 2022; 111:26–52. 10.1016/j.matbio.2022.05.003 35537652

[53] Wright CJ, Smith CWJ, Jiggins CD. Alternative splicing as a source of phenotypic diversity. Nat Rev Genet. 2022; 23:697–710. 10.1038/s41576-022-00514-4 35821097

[54] Li F, Xiong Y, Yang M, Chen P, Zhang J, Wang Q, Xu M, Wang Y, He Z, Zhao X, Huang J, Gu X, Zhang L, et al. c-Mpl-del, a c-Mpl alternative splicing isoform, promotes AMKL progression and chemoresistance. Cell Death Dis. 2022; 13:869. 10.1038/s41419-022-05315-5 36229456 PMC9561678

[55] Shao FF, Chen BJ, Wu GQ. The functions of EZH2 in immune cells: Principles for novel immunotherapies. J Leukoc Biol. 2021; 110:77–87. 10.1002/JLB.1RU0520-311R 33040370

[56] Li D, Wang HL, Huang X, Gu X, Xue W, Xu Y. Identification and Functional Characterization of a New Splicing Variant of EZH2 in the Central Nervous System. Int J Biol Sci. 2019; 15:69–80. 10.7150/ijbs.28129 30662348 PMC6329929

[57] Tolomeo M, Cascio A. The Multifaced Role of STAT3 in Cancer and Its Implication for Anticancer Therapy. Int J Mol Sci. 2021; 22:603. 10.3390/ijms22020603 33435349 PMC7826746

[58] Zhang W, Chu X, Tong Q, Cheung JY, Conrad K, Masker K, Miller BA. A novel TRPM2 isoform inhibits calcium influx and susceptibility to cell death. J Biol Chem. 2003; 278:16222–9. 10.1074/jbc.M300298200 12594222

[59] Tavares R, Wajnberg G, Scherer NM, Pauletti BA, Cassoli JS, Ferreira CG, Paes Leme AF, de Araujo-Souza PS, Martins-de-Souza D, Passetti F. Unveiling alterative splice diversity from human oligodendrocyte proteome data. J Proteomics. 2017; 151:293–301. 10.1016/j.jprot.2016.05.023 27222040

