# Supplementary material for: RNA splicing regulator EIF3D regulates the tumor microenvironment through immunogene-related alternative splicing in head and neck squamous cell carcinoma
Source: Aging (Albany NY). 2024 Mar 25;16(7):5929–48. doi: 10.18632/aging.205681 (PMC11042944; doi:10.18632/aging.205681)
Supplement: Supplementary Figures [file aging-16-205681-s001.pdf]

SUPPLEMENTARY FIGURES

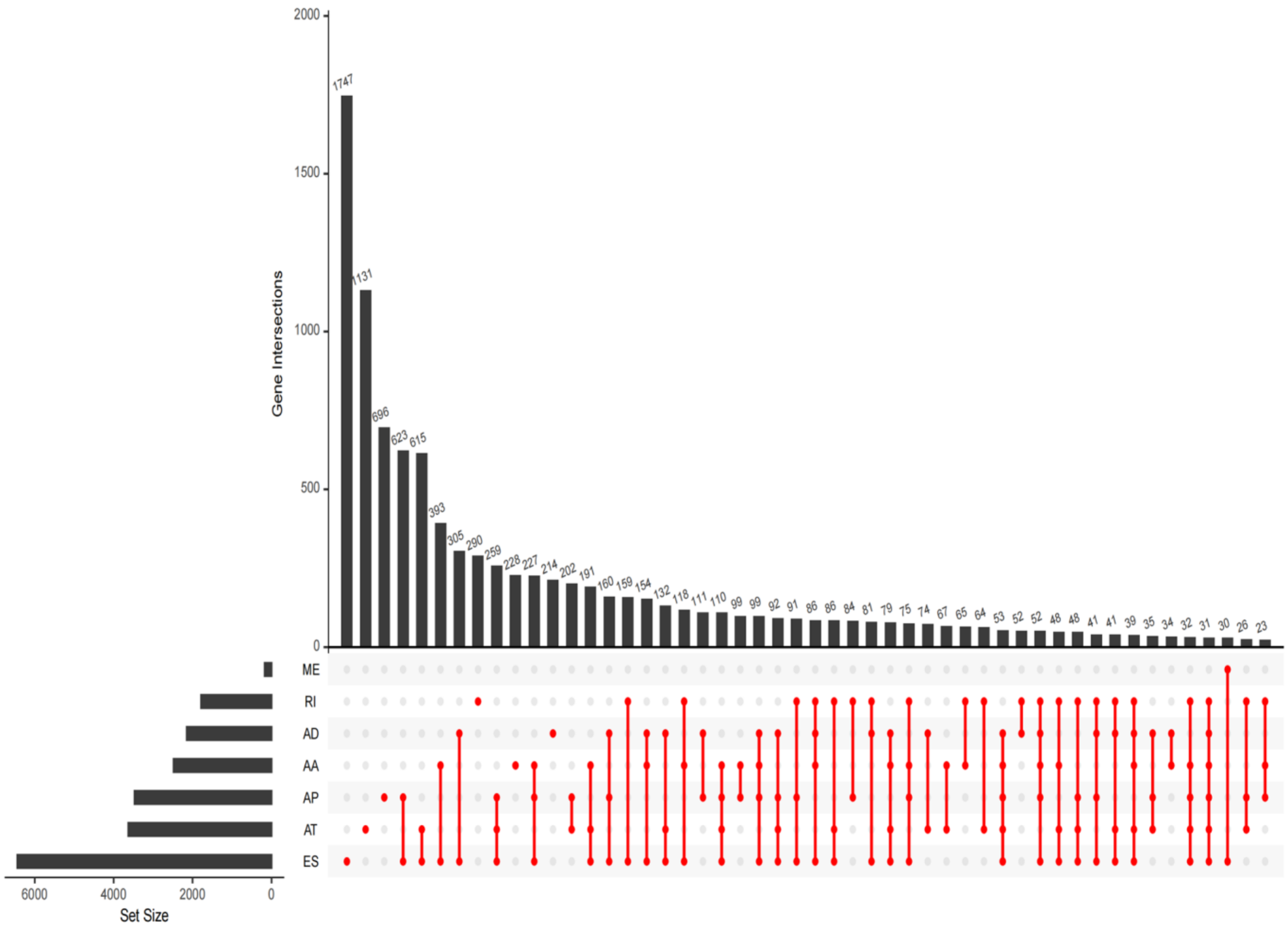

Supplementary Figure 1. UpSet plot illustrating interactions among the seven AS types detected for all parent genes of HNSC.

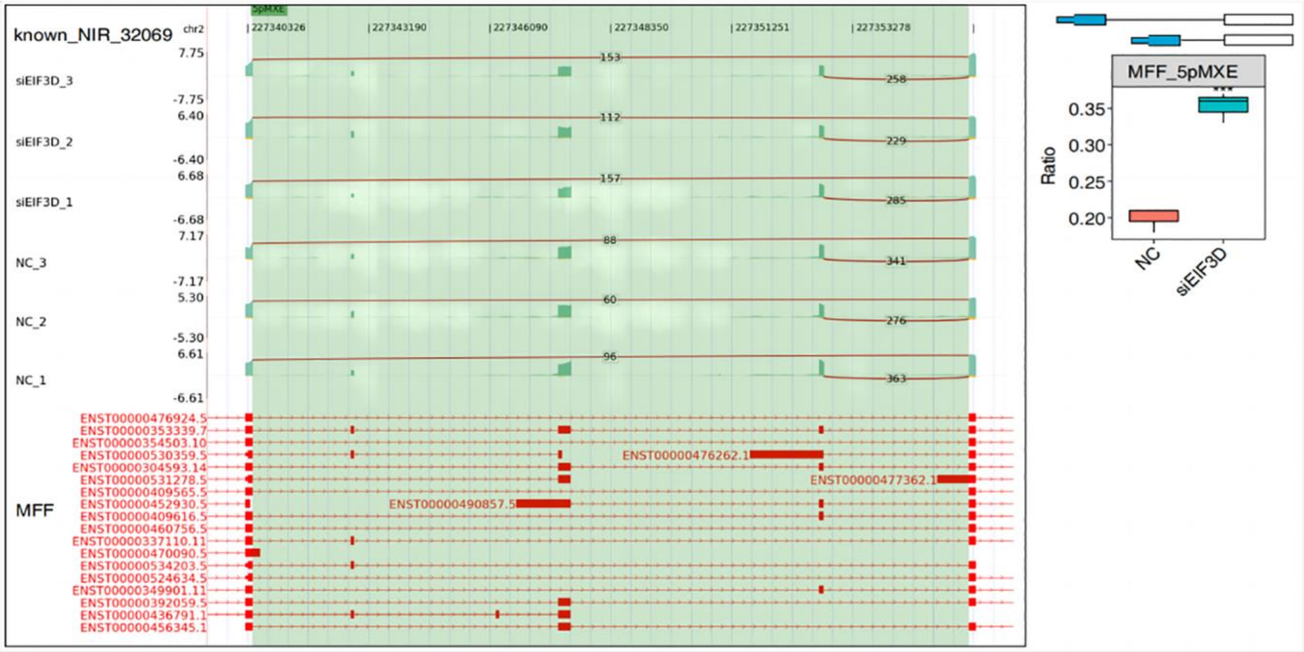

Supplementary Figure 2. Schematic diagrams depicting the structures of ASEs. RNA-seq validation of ASEs is displayed at the bottom of the right panel.

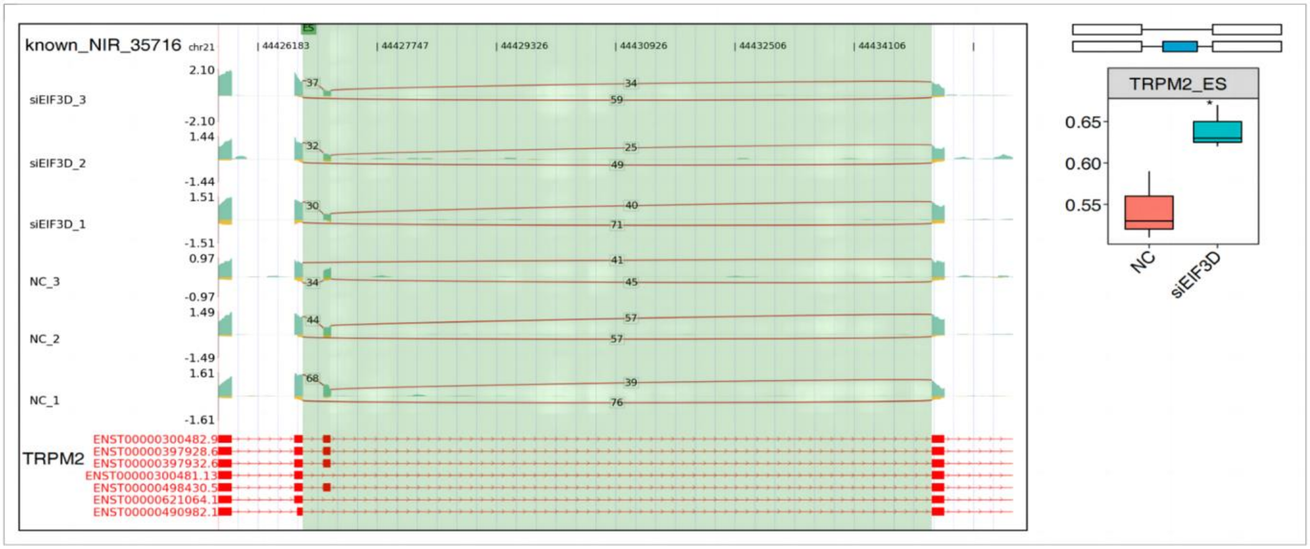

Supplementary Figure 3. Schematic diagrams depicting the structures of ASEs. RNA-seq validation of ASEs is displayed at the bottom of the right panel.
