# Supplementary material for: RNA splicing regulator EIF3D regulates the tumor microenvironment through immunogene-related alternative splicing in head and neck squamous cell carcinoma
Source: Aging (Albany NY). 2024 Mar 25;16(7):5929–48. doi: 10.18632/aging.205681 (PMC11042944; doi:10.18632/aging.205681)
Supplement: Supplementary Table 1 [file aging-16-205681-s002.pdf]

SUPPLEMENTARY TABLE

Supplementary Table 1. RT-qPCR primers for gene expression qualification.

Gene	Primer	Sequence (5′–3′)
GAPDH	Forward	GGTCGGAGTCAACGGATTTG
	Reverse	GGAAGATGGTGATGGGATTTC
EIF3D	Forward	CCAACAAGCAGGTCATCC
	Reverse	TTCCTCTTCTTCTTCCTCTTC
